# Metformin drugs under simulated gastric conditions can generate high nitrite-dependent levels of *N*-nitrosodimethylamine

**DOI:** 10.1038/s41598-024-63032-9

**Published:** 2024-06-17

**Authors:** George Cristian Georgescu, Mircea Cretu-Stancu, Octavian Bucur

**Affiliations:** 1 Victor Babes National Institute of Pathology, Bucharest, Romania; 2 Carol Davila University of Medicine and Pharmacy, Bucharest, Romania; 3 Dobrogen Tech, Ovidiu, CT Romania; 4 Genomics Research and Development Institute, Bucharest, Romania; 5 Viron Molecular Medicine Institute, Boston, MA USA

**Keywords:** *N*-nitrosodimethylamine (NDMA), *N*-nitrosodiethylamine (NDEA), Metformin, Acceptable intake (Ai), Active pharmaceutical ingredient-nitrite interaction, Internal standard, Diseases, Gastroenterology, Medical research, Risk factors

## Abstract

*N*-nitrosodimethylamine (NDMA) and *N*-nitrosodiethylamine (NDEA), group 2A carcinogens, were detected in finished drug products, including metformin, ranitidine, sartans and other drugs which caused multiple recalls in the USA and Europe. Important studies also reported the formation of NDMA when ranitidine and nitrite were added to simulated gastric fluid. Our objective was to screen finished drug products from Europe and USA for nitrosamine impurities and investigate the formation of NDMA in metformin finished drug products when added to simulated gastric fluid. One dosage unit of 30 different commercially available drugs, including metformin, sartans, and ranitidine were tested for NDMA, NDEA, and dimethylformamide (DMF) impurities, using a liquid chromatography-mass spectrometry (LC–MS) method. Then, 6 metformin finished drug products were tested in stomach conditions for 2 h at 37 °C in a 100 mL solution with a pH of 2.5 and different nitrite concentrations (40, 10, 1, 0.1 mM) and tested for NDMA, and DMF using LC–MS. We measured NDMA, NDEA, and DMF in 30 finished drug products. NDMA and DMF were quantified for metformin drug products in simulated gastric fluid with different nitrite concentrations. None of the 30 drugs showed concerning levels of NDMA, NDEA, or DMF when tested as single tablets. However, when metformin tablets are added to simulated gastric fluid solutions with high nitrite concentrations (40 mM and 10 mM), NDMA can reach amounts of thousands of nanograms per tablet. At the closest concentration to physiologic conditions we used, 1 mM, NDMA is still present in the hundreds of nanograms in some metformin products. In this in vitro study, nitrite concentration had a very important effect on NDMA quantification in metformin tablets added to simulated gastric fluid. 1 mM nitrite caused an increase above the acceptable daily intake set by the U.S. Food and Drug Administration (FDA) for some of the metformin drugs. 10 mM, 40 mM nitrite solutions generated NDMA amounts exceeding by more than a hundred times the acceptable daily intake set by the FDA of 96 nanograms. These findings suggest that metformin can react with nitrite in gastric-like conditions and generate NDMA. Thus, patients taking metformin could be exposed to NDMA when high nitrite levels are present in their stomach, and we recommend including a statement within the Patient Package Inserts/Instructions for use.

## Introduction

Nitrosamines are common contaminants in low amounts in many consumer goods from water to foods to tobacco smoke^1^. However, these products are generally exceptionally low in nitrosamine content. Based on animal studies^1–3^, exposure to *N*-nitroso-dimethylamine (NDMA) or *N*-nitroso-diethylamine (NDEA) is acutely toxic and chronically carcinogenic. Likewise, the common organic solvent N, *N*-Dimethylformamide (DMF) is known to be teratogenic in animals and was involved in the formation of NDMA^4^.

Although there is a limited number of conclusive studies in humans on the matter, NDMA, NDEA, and DMF are classified by the International Agency for Research on Cancer in group 2A (probable human carcinogen)^5^.

In 2018, angiotensin receptor blockers (ARB) joined the list of nitrosamine impurities sources when some valsartan^6^ and losartan^7^drug products were found contaminated with NDMA or NDEA, and recalled. Approximately 15 million patients^8^ had a written prescription for losartan or valsartan finished drug products at the time of the recall.

After ARB recalls, in 2019 and 2020, reports showed unacceptable nitrosamine levels in over-the-counter preparations of ranitidine^9^ and metformin^10^. While metformin was voluntarily recalled by some drug manufacturers^11^, the U.S. Food and Drug Administration (FDA) ordered the removal of all ranitidine preparations^12^. More than 20 million patients^8^ were taking metformin and over 1 million patients^8^ had a prescription for ranitidine in 2020 in the United States.

Similar measures were taken by the European Medicines Agency (EMA), i.e., suspending all ranitidine products in the European Union^13^ and asking manufacturers to test sartan and metformin drug products for nitrosamine impurities^14^ before releasing them on the market.

To assess the effectiveness of these measures within the European and American drug markets, we tested different commercially available, and post-market drug products. Investigated drugs included 13 metformin products, 17 sartan products, 1 injectable ranitidine product, 1 calcium-channel blocker, 1 anti-Parkinson agent, 1 antimicrobial agent, and 1 diuretic. The impurities we searched for were NDMA, NDEA, and DMF.

Structural similarities to ranitidine^15^ e. g. the dimethylamine group, and the proven reaction between DMA and nitrosating agents^16^ led us to test the potential of metformin finished drug products to generate NDMA in simulated gastric fluid.

## Methods

Commercially available finished drug products from Europe/Romania (30) were tested for NDMA, NDEA, and DMF as single tablets. Metformin finished drug products were evaluated for NDMA production and DMF in simulated gastric fluid. We tested various available metformin drugs with several concentrations of sodium nitrite, such as 40 mM, 10 mM, 1 mM, and 0.1 mM, in stomach conditions, including a few metformin drugs from the USA market, as specified in the Results section.

### Chemicals and reagents

Two nitrosamines (NDMA, NDEA), and DMF were selected as target analytes in this study. Reference standards of NDMA, NDEA, and isotope-labeled DMF standard D_7_-DMF were purchased from Sigma-Aldrich (St. Louis, MO). Isotopically labeled NDMA standard 13 C_2_-D_6_-NDMA was purchased from Cambridge Isotope Laboratories (Tewksbury, MA). Formic acid (LC–MS grade) was purchased from Millipore Sigma (Burlington, MA). Methanol (LC–MS grade) was purchased from Honeywell (Charlotte, NC). Milli-Q water (18.2 MΩ) was generated from a Milli-Q IQ Element system from Millipore Sigma.

### Sample preparation

The single drug tablet was accurately weighed. After the methanol dilution to approximately 100 mg active pharmaceutical ingredient (API) per mL of methanol, samples were homogenized. The last tube of rinsing methanol was used as a laboratory reagent blank (LRB) tested with the drug samples. A quality control positive (QCP) sample is also prepared from a recalled lot of Valsartan. Further dilution to 25 mg API/mL is achieved with Milli-Q water, followed by vortexing, centrifugation, and filtration through a 0.2 µm nylon filter to obtain a sample extract that was spiked with an isotopic internal standard mixture containing 666 ng of ^13^C_2_-D_6_-NDMA and 333 ng of D_7_-DMF. The vial subjected to the LC-HRMS contains a sample of 500 µL with 26 ng/mL ^13^C_2_-D_6_-NDMA and 13 ng/mL D_7_-DMF. The final internal standard concentration in each sample is 40 ng/mL.

For stomach conditions testing, metformin tablets were kept for 2 h at 37 °C and pH 2.5 in 100 mL solutions with 4 different sodium nitrite concentrations: 0.1 mM, 1 mM, 10 mM, and 40 mM.

### Instrumental analysis

SCIEX ExionLC AD (LC) coupled with SCIEX X500R quadrupole time-of-flight high resolution mass spectrometry (QToF HRMS) was purchased from SCIEX (Framingham, MA). The separation of the two nitrosamines and DMF is performed on an InfinityLab Poroshell 120 EC-C18, 4.6 × 100 mm, 2.7 µm analytical column (Agilent Technology, Santa Clara, CA).

Determination of target analytes is performed by SCIEX X500R QToF HRMS operated in positive atmospheric pressure ionization (APCI+) mode for the ionization of NDMA, NDEA, DMF and their isotopic labeled internal standards.

For a comprehensive description of the LC and HRMS parameters consult the supplemental data and the methods described within the reference^17^.

### Quality assurance and quality control (QA and QC)

All calibration and QC samples are prepared in 25/75 (v/v) methanol/water, following a protocol previously described in detail here^15^.

### Statistical analysis

Data are presented as mean and standard deviation (SD) of 3 independent experiments, unless specified otherwise. Data were collected and analyzed using Excel (Microsoft Corp).

## Results

### Commercial drug analysis for NDMA, NDEA, and DMF impurities

30 drugs from the European market were tested for NDMA, NDEA, and DMF impurities (Table 1). The main analyte, NDMA, was detected only in an injectable preparation of ranitidine and this was below the acceptable daily intake set by the FDA of 96 nanograms^18^. NDEA was not present in any of the drugs. However, DMF was present in most metformin preparations.

**Table 1:** Chemical impurity analysis of the first 30 finished drug products from the European Market.

Commercial name, active pharmaceutical ingredient, strength	NDMA (ng/dosage unit)	NDEA (ng/dosage unit)	DMF (ng/dosage unit)
Atacand, Candesartan cilexitil, 8 mg	ND	ND	< LOQ (41.1)
Candesartanum Cilexitil MCC 8 mg	ND	ND	< LOQ (41.5)
Canzeno,Candesartan cilexitil, 16 mg	ND	ND	ND
Karbis,Candesartan cilexitil, 8 mg	ND	ND	ND
Tandesar,Candesartan cilexitil, 8 mg	ND	ND	ND
Aprovel, Irbesartan, 150 mg	ND	ND	ND
Irbesartan Teva 150 mg	ND	ND	56,6
Irbesartan Zentiva 150 mg	ND	ND	ND
Diovan, Valsartan, 160 mg	ND	ND	ND
Lorista, Losartan, 25 mg	ND	ND	ND
Micardis, Telmisartan, 80 mg	ND	ND	56.1
Telmisartan Actavis 40 mg	< LOQ (4.2)	ND	< LOQ (41.8)
Tezeo, Telmisartan, 40 mg	ND	ND	ND
Tolura, Telmisartan 40 mg	ND	ND	ND
Micardisplus, Telmisartan/Hydrochlorothiazide, 80 mg/12.5 mg	ND	ND	ND
Telmisartan Hidroclorotiazida Egis 40 mg/12.5 mg	ND	ND	ND
Tezeo HCT, Telmisartan/Hydrochlorothiazide, 80 mg/12.5 mg	ND	ND	ND
Nefrix, Hydrochlorothiazide, 25 mg	ND	ND	ND
Levodopa/Carbidopa/Entacapona TEVA 100/25/200 mg	ND	ND	ND
Arnetin, Ranitidine, 50 mg/2 ml	5.9	ND	ND
Nitrofurantoina, Nitrofurantoin, Arena 100 mg	ND	ND	81.6
Leridip,Lercanidipine Hydrochloride, 10 mg	< LOQ (4.1)	ND	< LOQ (41.4)
Metfogamma, Metformin Hydrochloride, 1000 mg	< LOQ (22.5)	ND	973.1
Glucovance, Metformin Hydrochloride/Glibenclamide, 500 mg/2.5 mg	< LOQ (10)	ND	< LOQ (100.5)
Glibomet,Metformin Hydrochloride/Glibenclamide, 400 mg/2.5 mg	< LOQ (9.9)	ND	5847.9
Siofor, Metformin Hydrochloride, 1000 mg	ND	ND	19,266.5
Meguan, Metformin Hydrochloride, 500 mg	< LOQ (10)	ND	10,610.1
Metformin Aurobindo, Metformin Hydrochloride, 1000 mg	ND	ND	< LOQ (201.3)
Metformina Arena, Metformin Hydrochloride, 500 mg	ND	ND	8960.7
Glucophage XR, Metformin Hydrochloride, 500 mg	ND	ND	417.4

Results for one sample. *ND* Not Detected, *LOQ* Limit of Quantification, value given when the analyte is detected but not in amounts high enough to quantify.

### Metformin drugs in simulated gastric fluid

A previous study investigated the formation of NDMA from ranitidine in gastrointestinal conditions when reacting with nitrite in a low pH medium^19^.

The presence of the dimethylamine group in the molecule of the biguanide, metformin, and the proven interaction between nitrites and DMA to generate NDMA led us to evaluate metformin’s potential to form NDMA in a simulated gastric environment.

**Figure 1:**
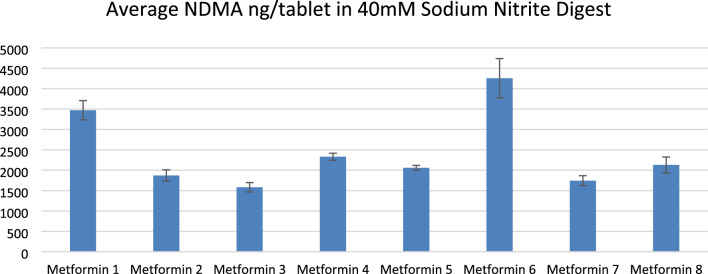
NDMA formation from metformin at pH 2.5 in simulated gastric fluid and 40 mM sodium nitrite concentration. A plot of the amount of NDMA at 2.5 pH in 100 mL simulated gastric fluid with 8 different metformin tablets and 40 mM sodium nitrite after 2 h incubation at 37 °C. The data represent the mean and SD of 3 independent samples, and the error bars show the SD of the measurement. The drugs are presented on the x-axis.

8 metformin tablets were dissolved in a stomach-like fluid solution (100 mL solution with a pH of 2.5 at 37 °C and in which drugs are kept for 2 h) containing 40 mM sodium nitrite and then analyzed using the HPLC–MS method. Results are summarized in Fig. 1. All tested drugs in these conditions show NDMA levels in the thousands of nanograms per tablet, amounts which easily exceed the acceptable daily intake established by the FDA of 96 nanograms. However, a concentration of 40 mM is also hard to reach in the stomach, in vivo. We further decided to test various available metformin drugs with lower concentrations of sodium nitrite, such as 10 mM, 1 mM, and 0.1 mM, in stomach conditions. In order to assess products from both markets, we selected 2 of the metformin drugs from Europe (shown in Fig. 1), one with the highest concentration of NDMA produced with 40 mM nitrite and a random one from the remaining 7 and 4 additional commercially available metformin drugs from the USA.

**Figure 2:**
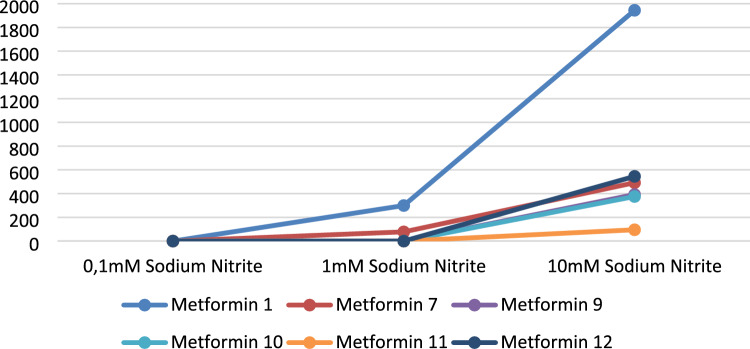
*N*-Nitrosodimethylamine formation from metformin at pH 2.5 in simulated gastric fluid and in increasing nitrite concentrations 0.1, 1, 10 mM. A plot of the variable NDMA amount at 2.5 pH in 100 mL simulated gastric fluid with different metformin tablets and 10 mM, 1 mM, and 0.1 mM sodium nitrite after 2 h incubation at 37 °C. The data is presented as the mean of 3 independent samples. For the data with mean and SD of 3 independent samples see Table 2.﻿

In Fig. 2, we present the data from the stomach conditions testing with 3 different nitrite concentrations. At 0.1 mM sodium nitrite, no NDMA was detected in any of the 6 drugs. 1 mM nitrite caused an increase above the acceptable daily intake set by the U.S. Food and Drug Administration (FDA) for some of the metformin drugs, while the 10 mM nitrite and higher concentrations led to NDMA amounts way above the acceptable daily intake set by FDA of 96 nanograms.

.

**Table 2:** The data represents the mean and standard deviation of 3 independent samples.

	1 mM		10 mM	
	Average ng/tablet	stdev ng/tablet	Average ng/tablet	stdev ng/tablet
Metformin 2	77.59300073	24.50410808	491.7418019	139.0811703
Metformin 6	299.485741	18.78575381	1944.176184	160.1452946
Metformin 9	ND	–	392.1767515	93.77949319
Metformin 10	ND	–	375.3589795	18.09369911
Metformin 11	ND	–	95.24143458	17.51835379
Metformin 12	ND	–	545.0210379	33.61993931

0.1 mM column is not shown since the values were ND. *ND* Not Detected.

## Discussion

Various reasonable and common situations exist in which concentrations of nitrite could be significantly elevated. As an example, consuming one pound of dry-cured meat that contains the regulatory limit of nitrite (625 ppm)^20^ would bring 800 mL of stomach volume to a nitrite concentration of over 5 mM.

All drugs tested in the presence of 40 mM of sodium nitrite in stomach conditions show NDMA levels in the thousands of nanograms per tablet, amounts which significantly exceed the acceptable daily intake established by the FDA of 96 nanograms. For example, Metformin 6 and Metformin 1 have 4255 ng/tablet and 3470 ng/tablet of NDMA, respectively. However, a concentration of 40 mM is also hard to reach in the stomach, in vivo. Thus, we repeated the experiment with 2 selected metformin drugs and 4 metformin drugs from the USA market at lower concentrations of sodium nitrite, namely 0.1 mM, 1 mM, and 10 mM. Metformin 6 showed the highest NDMA amount per tablet, and Metformin 11 the lowest.

## Limitations

The in vitro model used in this study has inherent limitations and does not reflect all aspects of human physiology. However, it does provide a method for assessing the potential for physiologic nitrite reactions with drugs in the gastric fluid that lead to NDMA formation.

## Conclusion

In this in vitro study, nitrite concentration had an especially important effect on NDMA quantification in metformin tablets added to simulated gastric fluid. 1 mM nitrite caused an increase above the acceptable daily intake set by the U.S. Food and Drug Administration (FDA) for some of the metformin drugs, while the 10 mM nitrite and higher concentrations led to NDMA amounts exceedingly elevated compared to the acceptable daily intake set by the FDA of 96 nanograms. These findings suggest that metformin can react with nitrite in gastric-like conditions and generate NDMA. Thus, patients taking metformin could be exposed to NDMA, when high nitrite levels are present in their stomach, therefore leading to exposure to higher-than-normal concentrations of NDMA. This metformin-nitrite interaction in the gastric medium is even more relevant when considering how the biguanide is prescribed i.e. it should be taken with food^21^. Further studies are needed to evaluate the interaction between nitrite content of the meal and its interaction in the gastrointestinal tract with metformin or its degradation product, DMA.

### Supplementary Information

Supplementary Information.

## Data Availability

The datasets used and/or analysed during the current study are available from the corresponding author on reasonable request.
