# Supplementary material for: Metformin drugs under simulated gastric conditions can generate high nitrite-dependent levels of N-nitrosodimethylamine
Source: Sci Rep. 2024 Jun 17;14:13910. doi: 10.1038/s41598-024-63032-9 (PMC11183255; doi:10.1038/s41598-024-63032-9)
Supplement: Supplementary file 1 — Supplementary Information. [file 41598_2024_63032_MOESM1_ESM.docx]

| Paper Name | Distributor | Lot number |
| --- | --- | --- |
| Metformin 1 | Wörwag | 19J215 |
| Metformin 2 | Merck | Y2785 |
| Metformin 3 | Berlin-Chemie | 8068.00 |
| Metformin 4 | Berlin-Chemie | 15004.00 |
| Metformin 5 | Gedeon | F0C022A |
| Metformin 6 | Aurobindo | FNM20048-10A |
| Metformin 7 | Arena | 337112020 |
| Metformin 8 | Merck | E206841 |
| Metformin 9 | Bayshore | 20942 |
| Metformin 10 | Granules | 4910206A |
| Metformin 11 | Sun | JKX3507A |
| Metformin 12 | Viona | M011304 |

**Supplementary Table 1. Company and lot number of the metformins used within the manuscript**
